# Ensuring Integrity in Comparative Effectiveness Research: Accentuate the Negative

**DOI:** 10.1371/journal.pmed.1000152

**Published:** 2009-09-29

**Authors:** The PLoS Medicine Editors

## Abstract

The *PLoS Medicine* Editors discuss the importance of negative studies in maintaining the integrity of the medical literature, especially when clinical effectiveness at issue.

In recent weeks, *PLoS Medicine* has published several research papers that challenge current health care practices.

One study found that a campaign to promote solar drinking water disinfection did not substantially decrease rates of childhood diarrhea [1]. Another found more than a doubled risk of hospitalization for bradycardia in older people taking cholinesterase inhibitors used to treat cognitive impairment [2]. A systematic review failed to find any randomized trials that support the internationally recommended retreatment regimen for tuberculosis [3]. Finally, a study of one of the main international registries for clinical trials, ClinicalTrials.gov, found that requirements to register clinical trials have not resulted in high publication rates [4].

A study that questions the accepted or desired way of doing things can be at least as important as one that supports a new approach. Physicians mindful of Hippocrates' vow to “first do no harm” should take a keen interest in such studies, for while a new intervention that proves efficacious in clinical trials may pass slowly, if at all, through practical barriers to effective implementation, the demonstration that an existing practice is ineffective or potentially harmful can (or should) prompt a rapid change in research agendas, policy, and clinical care. Knowing what doesn't work is particularly useful in efforts to control medical spending, where redirecting limited resources away from ineffective interventions is of obvious benefit.

Studies that convincingly refute accepted practice are particularly well suited to open-access publication. Only universal availability of unexpected or, from a marketing standpoint, unfavorable data can effectively guard against publication bias that may favor results that advance specific vested interests. Such bias can skew the medical literature toward optimistic but incomplete data and can prevent consideration of the full evidence in policy-changing systematic reviews. Open-access journals are, by definition, free from the temptation to reap profits by selling reprints of research papers, and therefore need have no reservations about publishing papers that, by failing to promote a new intervention, create little commercial demand for reprints. Open-access journals also need not be concerned if negative efficacy studies don't receive the kind of media coverage that attracts paying readers in large numbers.

The importance of valid negative results, and of their immediate and open availability, is of particular pertinence in the movement to undertake comparative effectiveness research (CER), which earlier this year received US$1.1 billion in United States government funding [5]. In a report commissioned by Congress as part of the American Recovery and Reinvestment Act, the Institute of Medicine (IOM) defined comparative effectiveness research as “the generation and synthesis of evidence that compares the benefits and harms of alternative methods to prevent, diagnose, treat, and monitor a clinical condition or to improve the delivery of care. The purpose of CER is to assist consumers, clinicians, purchasers, and policy makers to make informed decisions that will improve health care at both the individual and population levels.” In setting priority areas for comparative effectiveness research, the IOM report noted that “priorities should be balanced in the primary methodologies employed to conduct them: systematic reviews, database research, observational studies, and randomized trials”[6].

In calling for a wide range of study designs to assess real-world outcomes, this initiative raises the need for broad transparency in the analysis and reporting of study results. In recent years, journals and governments have recognized the need for openness in the registration and reporting of clinical trials [7], which are designed primarily to determine safety and efficacy. In CER, which will include studies of many types, practices that distort the scientific evidence base—such as “cherry picking” for publication only those studies describing a desired outcome, or “fishing” from an ocean of possible analyses only those that might support favorable (but statistically invalid) conclusions—have the potential to affect policy, practice, and profits to an even greater degree than they have done in the context of traditional efficacy trials. Registering only clinical trials is therefore insufficient to protect against selective publication or reporting in studies of other designs, which will play an increasing role in assessing clinical effectiveness. In other words, a journal that adheres to now-standard requirements relating to trial registration might duly intercept an unregistered clinical trial of an abandoned intervention that will not affect care, but might have no way of knowing that a large data-mining study intended to influence treatment guidelines is presenting a non-prespecified (“post-hoc”) analysis as its primary outcome. As the IOM report observes:

“Objectivity will be central to the public's trust and confidence in the integrity of the CER Program. Conflict of interest and bias in clinical research—published in even the most respected medical journals—is well-documented…. Selective reporting or publication bias is common. Positive findings are more likely to be published than negative results…. In addition, there have been significant instances in which leading journals have not sufficiently enforced disclosure requirements for authors and reviewers…. CER is as vulnerable to bias and conflict of interest as any other area of medical research. The ultimate value of the CER enterprise will rest, in part, on vigilant attention to these issues” [6].

CER thus provides a wake-up call that draws attention to the potential pitfalls of bias in research more generally, and highlights the need for openness in the design and reporting of all types of research, in order to prevent ulterior interests—whether financial or political—from slanting data in a manner that could directly affect the medical care of large populations. If studies of many designs, and those producing negative as well as positive assessments, are to play a larger role in shaping clinical practice, then transparency in the design and reporting of these studies will be essential. The affected public should demand prospective registration of all research—regardless of study design—that is aimed at assessing the effectiveness of clinical practices. Venues in which to begin are available; ClinicalTrials.gov already accommodates registration of observational as well as interventional studies. With heightened vigilance on the part of journal editors and policymakers, such registration, by laying out intended analyses in advance, can prevent studies from shape-shifting or simply vanishing without explanation between registration and publication, as seems to be happening with many clinical trials [4]. Moreover, reporting guidelines, such as those disseminated by the EQUATOR Network [8], are not merely aids for structuring manuscripts, but provide valuable tools for designing studies that are transparent to critical evaluation.

Medical practice stands to benefit from a clearer understanding of what works effectively and what doesn't. Such knowledge can only emerge from unbiased reporting of the research that supports it, including data that contest prevailing practices. The integrity of the medical literature requires that negative results assume their rightful place alongside those that support blockbuster sales and breakthrough headlines.

## Abbreviations

IOM: Institute of Medicine
CER: comparative effectiveness research
